# Resynchronization effects and clinical outcomes during left bundle branch area pacing with and without conduction system capture

**DOI:** 10.1002/clc.23969

**Published:** 2023-01-03

**Authors:** Weiwei Zhang, Lu Chen, Xiaohong Zhou, Jingjuan Huang, Shiwei Zhu, E. Shen, Changqing Pan, Xumin Hou, Ruogu Li, Ben He

**Affiliations:** ^1^ Department of Cardiology, Shanghai Chest Hospital, School of Medicine Shanghai Jiao Tong University Shanghai China; ^2^ Department of Ultrasound, Shanghai Chest Hospital, School of Medicine Shanghai Jiao Tong University Shanghai China; ^3^ Cardiac Rhythm and Heart Failure Division Medtronic plc Mounds View Minnesota USA; ^4^ Shanghai Chest Hospital, School of Medicine Shanghai Jiao Tong University Shanghai China

**Keywords:** cardiac resynchronization therapy, heart failure, left bundle branch area pacing, left bundle branch pacing, left ventricular conduction time, left ventricular septal myocardial pacing

## Abstract

**Background:**

Left bundle branch area pacing (LBBAP) includes left bundle branch pacing (LBBP) and left ventricular (LV) septal myocardial pacing (LVSP).

**Hypothesis:**

The study aimed to assess resynchronization effects and clinical outcomes by LBBAP in heart failure (HF) patients with cardiac resynchronization therapy (CRT) indications.

**Methods:**

LBBAP was successfully performed in 29 consecutive patients and further classified as the LBBP‐group (*N* = 15) and LVSP‐group (*N* = 14) based on the LBBP criteria and novel LV conduction time measurement (LV CT, between LBBAP site and LV pacing (LVP) site). AV‐interval optimized LBBP or LVSP, or LVSP combined with LVP (LVSP‐LVP) was applied. LV electrical and mechanical synchrony and clinical outcomes were assessed.

**Results:**

All 15 patients in the LBBP‐group received optimized LBBP while 14 patients in the LVSP‐group received either optimized LVSP (5) or LVSP‐LVP (9). The LV CT during LBBP was significantly faster than that during LVP (*p* < .001), while LV CT during LVSP were similar to LVP (*p* = .226). The stimulus to peak LV activation time (Stim‐LVAT, 71.2 ± 8.3 ms) and LV mechanical synchrony (TSI‐SD, 35.3 ± 9.5 ms) during LBBP were significantly shorter than those during LVSP (Stim‐LVAT 89.1 ± 19.5 ms, TSI‐SD 49.8 ± 14.4 ms, both *p* < .05). Following 17(IQR 8) months of follow‐up, the improvement of LVEF (26.0%(IQR 16.0)) in the LBBP‐group was significantly greater than that in the LVSP‐group (6.0%(IQR 20.8), *p* = .001).

**Conclusions:**

LV activation in LBBP propagated significantly faster than that of LVSP. LBBP generated superior electrical and mechanical resynchronization and better LVEF improvement over LVSP in HF patients with CRT indications.

## 1 INTRODUCTION

Left bundle branch area pacing (LBBAP) is considered as a physiological pacing modality that produces a relatively short electrocardiogram (ECG) QRS duration (QRSd), fast left ventricular (LV) activation and preserves LV synchrony. Several clinical investigations have shown procedural feasibility and favorable clinical outcomes by LBBAP in patients with heart failure (HF) and left bundle branch block (LBBB).^1^, ^2^, ^3^ Thus, LBBAP has recently been proposed as a strategy for cardiac resynchronization therapy (CRT).

LBBAP is achieved by placing the pacing lead tip in the left side of the interventricular septum (IVS) near the location of the left bundle branch (LBB). However, in routine clinical practice, two types of pacing modalities were observed, LBB pacing (LBBP) and left ventricular septal myocardial pacing (LVSP). LBBP directly recruits the cardiac conduction system and preserves the natural LV activation pattern or synchrony, while LVSP produces a slower LV activation without direct recruitment of the conduction system.^4^ The differences of electrical characteristics between LBBP and LVSP have been investigated.^5^, ^6^

Although LBBP has well been defined,^7^ direct LBB capture may not always be achievable, especially when the operator limits the number of lead screw‐in attempts or due to anatomical variation between patients. It remains unknown whether LVSP can provide clinical benefits similar to LBBP in HF patients. In the present study, we developed a practical technique to measure LV conduction time for assessing whether the pacing captured LBB or not. Therefore, the objectives of this study were to evaluate the therapeutic effects of LBBAP in HF patients with CRT indications and assess the electrical characteristics and mechanical resynchronization in LBBAP with a comparison between LBBP and LVSP.

## 2 METHODS

### 2.1 Patient selection

Twenty‐nine consecutive patients with symptomatic HF and CRT indications were prospectively enrolled from July 2019 to June 2021. All patients received standard medications for HF treatment for at least 3 months and conformed to the following inclusion criteria: LV ejection fraction (LVEF) ≤ 40%, QRSd ≥130 ms with LBBB or intraventricular conduction delay (IVCD).

The study protocol was approved by the hospital ethics committee and conformed with the Declaration of Helsinki. Informed consent has been obtained from each patient.

### 2.2 LBBAP procedure

LBBAP was achieved via the transventricular septal approach using the SelectSecure™ pacing lead (Model 3830, 69 cm, Medtronic Inc.) which was delivered through a fixed curve sheath (C315HIS, Medtronic Inc.) as described previously.^3^, ^7^ The 3830 pacing lead was screwed deeply through the IVS until it reached the left side of the IVS. The number of the lead screwing into the left side of IVS was limited to no more than five times.

LBBP was characterized with: (1) the paced ECG QRS morphology of right bundle branch conduction block (RBBB), (2) abrupt shortening (≥10 ms) of the stimulus to peak left ventricular activation time (Stim‐LVAT) in ECG lead V6 with an increasing pacing output or constant and shortest Stim‐LVAT both at high and low outputs, (3) in patients with non‐LBBB, the presence of LBB potential on electrogram (EGM) were also needed to confirm LBB capture.^2^, ^7^ If the characteristics of paced ECG and EGM did not meet the LBBP criteria despite the presence of a RBBB pattern in ECG lead V1, the pacing was classified as LVSP.

Atrial pacing lead, LV coronary sinus (CS) lead and right ventricular (RV) defibrillation lead (DF‐1 lead) were implanted according to the standard procedures. A bipolar LV lead (Attain Ability™, Medtronic Inc.) was positioned in a lateral or posterolateral coronary vein.

### 2.3 Device programming and pacing optimization

In two patients in whom the LV lead was not successfully implanted due to the severe distortion of coronary vein or phrenic nerve stimulation, a dual‐chamber pacemaker was implanted. For the patients with CRT‐D, the LBBAP lead was inserted to the RV IS‐1 connector port and the RV lead IS‐1 connector pin was capped. LBBAP was delivered using bipolar pacing.

**Figure 1:**
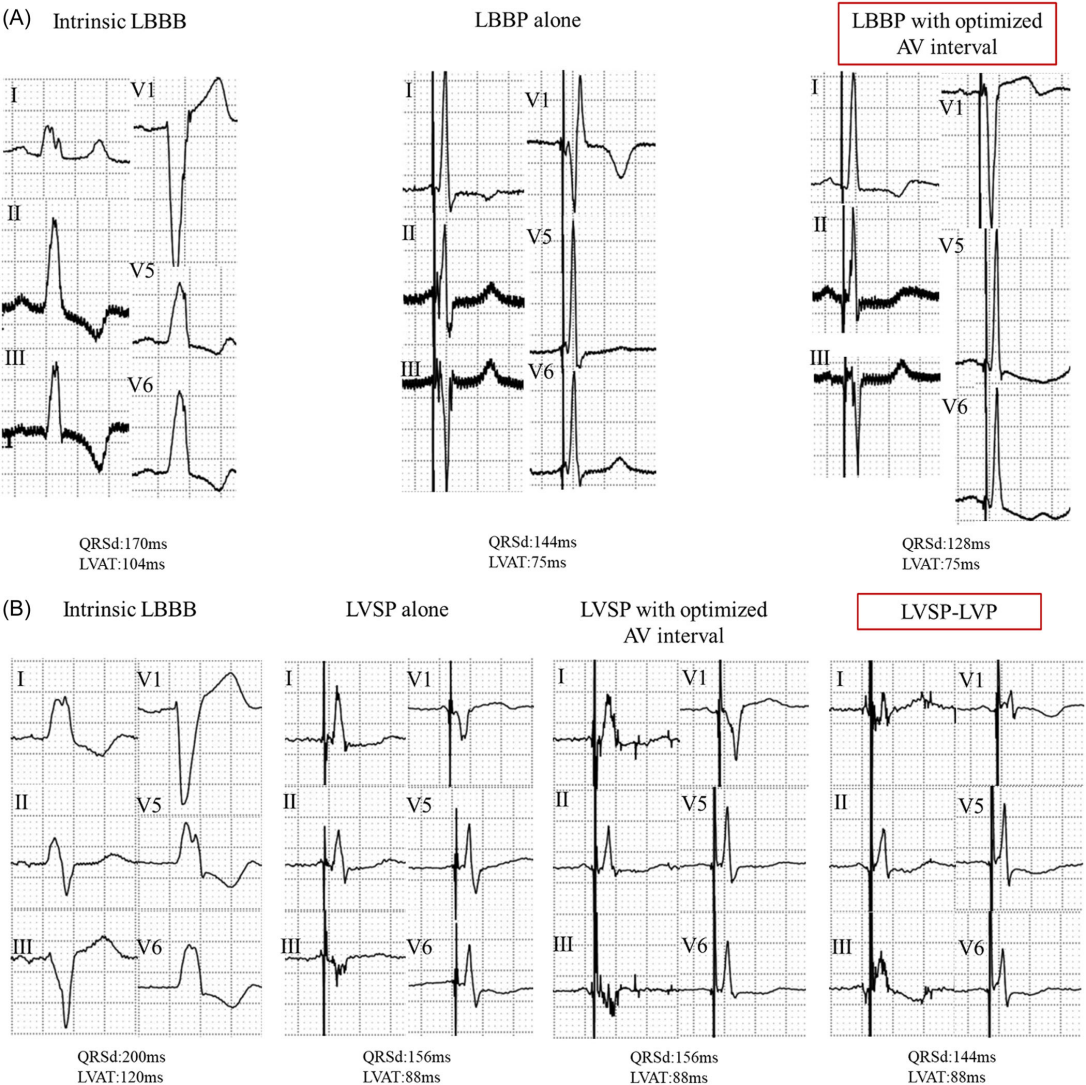
12‐lead ECGs during LBBAP optimization. (A) LBBP with optimized AV interval (120/150 ms following AV delay sweeping test) was chosen as the final pacing mode. (B) LVSP‐LVP achieved the shortest QRSd, and hence was chosen as the final pacing mode. ECG, electrocardiogram; LBBAP, left bundle branch area pacing; LBBB, left bundle branch block; LBBP, left bundle branch pacing; LVAT, peak left ventricular activation time; LVP, left ventricular pacing; LVSP, left ventricular septal myocardial pacing; QRSd, QRS duration.

After device implantation, 12‐lead ECGs were recorded during different pacing modes including LBBAP with optimized AV interval (optimized LBBAP with AV delay sweeping from 80 ms to the intrinsic PR interval to determine the optimal AV interval) and LBBAP combined with LV pacing (LBBAP‐LVP) based on V‐V delay sweeping from −40 to 40 ms. Among patients in whom LBBP was achieved, optimized LBBP with an AV interval that produced the shortest paced‐QRSd was applied. In patients who received LVSP, optimized LVSP and LVSP‐LVP, whichever produced the shortest paced‐QRSd was chosen as the final pacing mode (Figure 1).

### 2.4 Data collection and follow‐up

Baseline data before enrollment were collected, including demographics, medical history, HF‐related medications, plasma level of B‐type natriuretic peptide (BNP), New York Heart Association (NYHA) classification, ECG parameters, echocardiogram parameters, and HF hospitalization.

After device implantation, all patients were regularly followed up at 3 months, 6 months, and every 6 months thereafter. At follow‐up, the percentage of ventricular pacing (VP), pacing parameters, and 12‐lead ECG during pacing at different pacing outputs were collected. BNP, NYHA classification and HF hospitalization were also documented. Medications were adjusted according to patients' HF symptoms and were documented.

Echocardiogram was assessed using commercially available system (Vivid E95, GE Vingmed Ultrasound; 3.5‐MHz transducer). LVEF, LV end diastolic dimension (LVEDD), LV end systolic dimension (LVESD), left atrial dimension (LAD), mitral valve regurgitation (MR), and tricuspid valve regurgitation (TR) were measured at baseline and follow‐up. LVEF was estimated using the Simpsons' biplane method. At 6‐month follow‐up, interventricular mechanical synchrony and left intraventricular mechanical synchrony were evaluated in intrinsic rhythm, optimized LBBAP, and LBBAP‐LVP, respectively. Interventricular mechanical delay (IVMD) was measured by pulsed‐wave Doppler to evaluate interventricular mechanical synchrony. Tissue synchronization imaging (TSI) of 12 LV segments was used for assessing left intraventricular mechanical synchrony.^8^, ^9^

**Figure 2:**
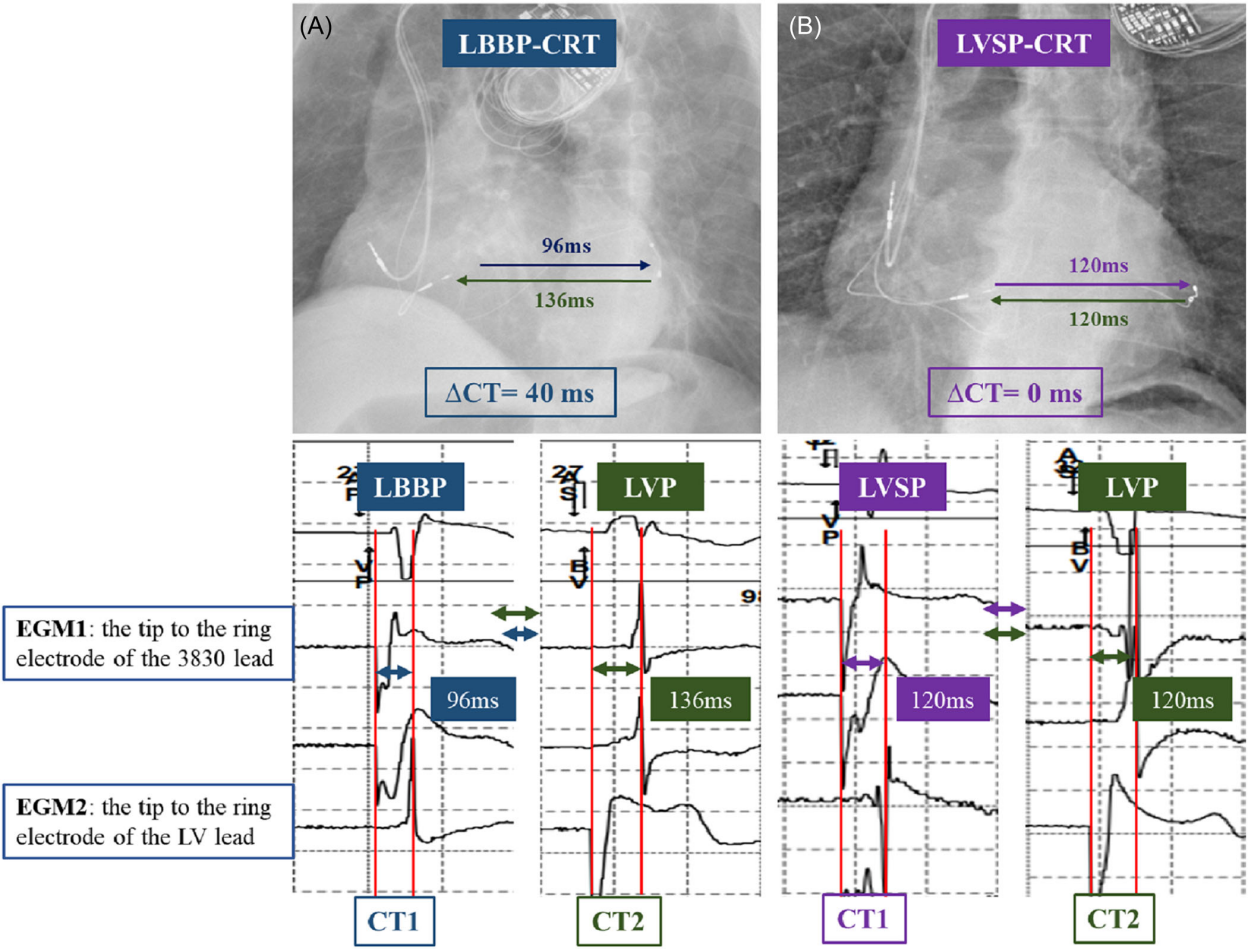
Measurement of left ventricular activation time during LBBAP and LVP to distinguish LBBP from LVSP. The conduction time (CT1) from LBBAP to the activation detected by the distal electrodes of LV lead was compared with the interval (CT2) from LVP to the activation detected by the LBBAP lead in LV septum. (A) During LBBP in one patient, the value of CT2 minus CT1 (∆*C*_T_) was 40 ms. (B) During LVSP in another patient, the value of CT2 minus CT1 (∆*C*_T_) was 0 ms. CRT, cardiac resynchronization therapy; EGM, electrogram, LBBAP, left bundle branch area pacing; LBBP, left bundle branch pacing; LV, left ventricular; LVP, left ventricular pacing; LVSP, left ventricular septal myocardial pacing.

At follow‐up, intracardiac EGM and 12‐lead ECG were simultaneously recorded at working outputs (3V/0.4 ms) of LBBAP and LVP in 27 patients with a LV CS lead. EGMs during LBBAP and LVP were analyzed to confirm LBBP or LVSP as following: The conduction time (CT1) from LBBAP to the activation detected by the distal electrodes of LV lead was compared with the interval (CT2) from LVP to the activation detected by the LBBAP lead electrodes (Figure 2). If the value of CT2 minus CT1 (∆*C*_T_) was ≥20 ms, LBBP was assumed because the paced activation propagation through the conduction system is faster (a shorter CT1) than that through the myocardial tissue (a longer CT2) (Figure 2A). If the ∆CT was <20 ms, then the LVSP was further confirmed because the activation propagation time without the conduction system direct capture was similar between the LVSP to the LVP site and the LVP to the LVSP site (Figure 2B).

Responses to pacing therapy were classified as super response (an absolute improvement in LVEF ≥ 15% or LVEF ≥ 45% at follow‐up), moderate response (5% ≤ an absolute improvement in LVEF < 15%), and nonresponse (an absolute improvement in LVEF < 5%).

### 2.5 Statistical analysis

Normally distributed continuous variables were expressed as mean ± SD. Between‐group comparisons and within‐group comparisons were made using independent‐sample *T*‐test and paired‐sample *T*‐test, respectively. Non‐normally distributed continuous variables were expressed as median (interquartile range, IQR) and were compared using the Mann–Whitney U‐test. Categorical variables were expressed as numbers and percentages, and were compared using the *χ* ^2^ test or Fisher's exact test. All tests were two‐tailed and *p* < .05 was considered statistically significant. All statistical analysis was carried out by SPSS 25 (IBM Corp.).

## 3 RESULTS

### 3.1 Baseline characteristics

Baseline characteristics of all patients are summarized in Supporting Information: Table S1. Among all 29 patients with CRT indications, 24 patients had LBBB and 5 were IVCD. The mean LVEF was 32.2 ± 5.1%, NYHA classification was 3 (0), intrinsic QRSd was 171.9 ± 14.5 ms and LVAT was 106.5 ± 25.0 ms. Twenty‐five of the 29 patients (86.2%) had nonischemic cardiomyopathy.

At implantation, LBBP was achieved in 16 patients (LBBP group) and LVSP was accepted in the remaining 13 patients (LVSP group). At early follow‐up, one patient with LBBP at implantation was considered to have LVSP because LVAT increased by >10 ms compared to that at implantation and the CT1 was similar to the CT2 (∆*C*_T_ < 20 ms). Thus, there were 15 patients in the LBBP group and 14 patients in the LVSP group at follow‐up. Of the 24 patients with LBBB, 9 patients were in the LVSP group while 15 were in the LBBP group (64.3% vs. 100.0%, *p* = .017). Five patients with IVCD were all in the LVSP group (*p* = .017 vs. the LBBP group). There were no significant differences in other baseline data between the two groups (Supporting Information: Table S1). All 15 patients in the LBBP group received optimized LBBP, while 5 of 14 patients in the LVSP group received optimized LVSP and the remaining 9 LVSP‐LVP.

### 3.2 Electrical characteristics of LBBAP

**Figure 3:**
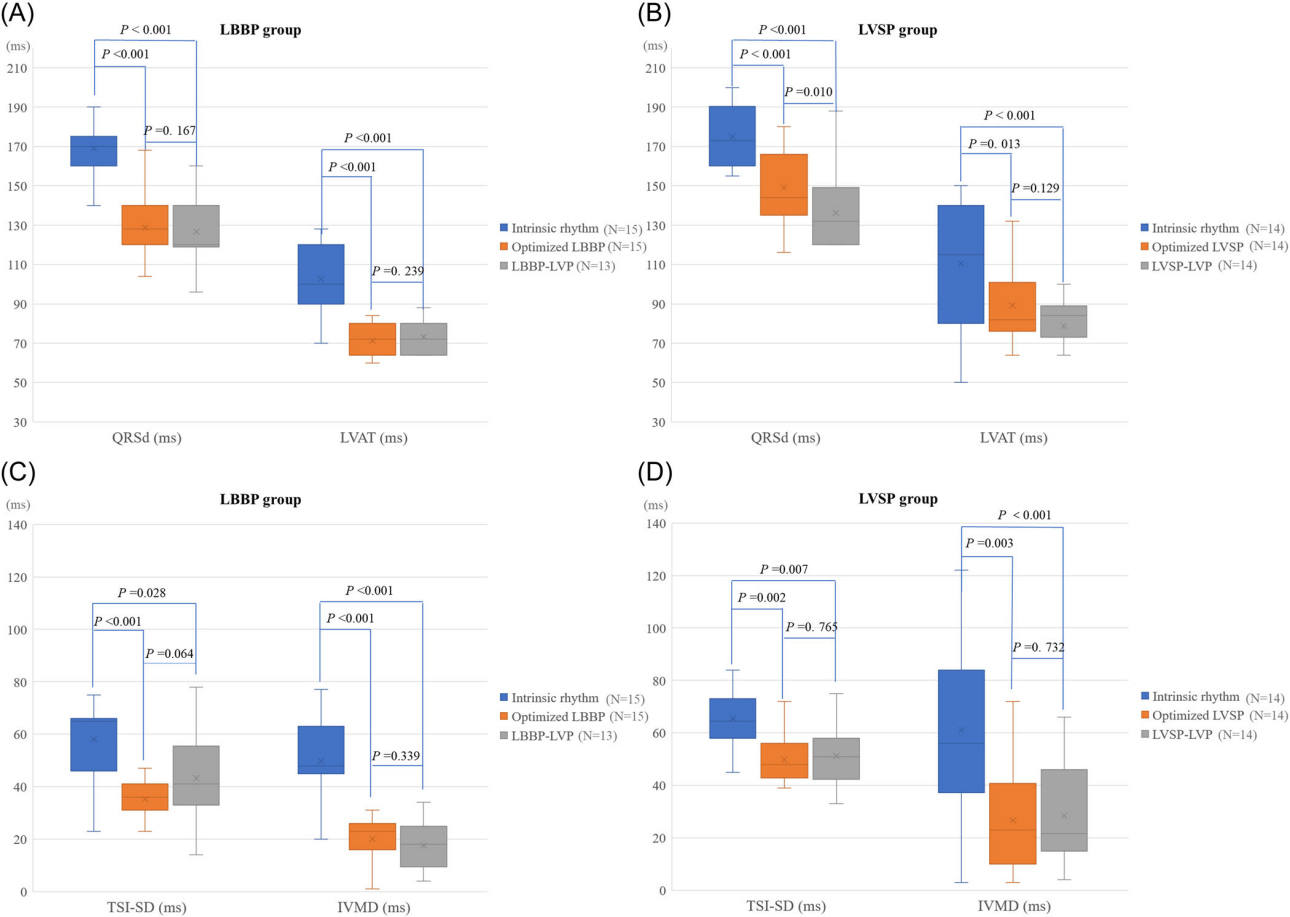
Electrical and mechanical synchrony in intrinsic rhythm and different pacing modes. (A–B) Paced‐QRSd and LVAT during intrinsic rhythm, optimized LBBAP and LBBAP‐LVP in the LBBP group (A) and the LVSP group (B). (C–D) The SD of tissue synchronization imaging of 12 LV segments (TSI‐SD) and interventricular mechanical delay (IVMD) during intrinsic rhythm, optimized LBBAP and LBBAP‐LVP in the LBBP group (C) and the LVSP group (D). The two patients without LV CS leads in the LBBP group were excluded when performing the paired comparisons of LBBP‐LVP with LBBP or intrinsic rhythm. CS, coronary sinus; LBBAP, left bundle branch area pacing; LBBP, left bundle branch pacing; LV, left ventricular; LVAT, peak left ventricular activation time; LVP, left ventricular pacing; LVSP, left ventricular septal myocardial pacing; QRSd, QRS duration.

ECG characteristics during intrinsic rhythm and different pacing modes at follow‐up were shown in Figure 3A,B and Supporting Information: Table S2. The paced‐QRSd and Stim‐LVAT during optimized LBBP were significantly shorter than those in intrinsic rhythm (both *p* ＜ .001), and also significantly shorter than those during optimized LVSP (both *p* = .005 vs. during LBBP, respectively). In patients with optimized LVSP, the paced‐QRSd and Stim‐LVAT were significantly shorter than those during intrinsic rhythm (*p* = .001, *p* = .013, respectively). However, LVSP‐LVP could further shorten paced‐QRSd and Stim‐LVAT (compared with those during optimized LVSP, *p* = .010, *p* = .129, respectively; compared with those in intrinsic rhythm, both *p* < .001).

In the LBBP group, the CT1 (85.8 ± 14.8 ms) was significantly shorter than the CT2 (116.3 ± 14.5 ms, *p* < .001) (Δ*C*_T_ = 30.5 ± 7.4 ms with all individual Δ*C*_T_ ≥ 20 ms). In the LVSP group, the CT1 (107.4 ± 24.1 ms) was similar to the CT2 (110.3 ± 22.5 ms, *p* = .226) (Δ*C*_T_ = 2.9 ± 9.2 ms, all individual Δ*C*_T_ ≤ 16 ms).

### 3.3 Echocardiographic mechanical synchrony during LBBAP

As shown in Figure 3C,D and Supporting Information: Table S2, there was no significant difference in TSI‐SD and IVMD under intrinsic rhythm between the two groups (*p* = .133, .242, respectively). Compared with intrinsic rhythm, both the pacing modes of optimized LBBAP and LBBAP‐LVP significantly shortened IVMD and TSI‐SD. TSI‐SD during optimized LBBP in the LBBP group was significantly smaller than that during optimized LVSP in the LVSP group (*p* = .003). In the LBBP group, TSI‐SD during the optimized LBBP trended smaller than that during LBBP‐LVP (*p* = .064). In the LVSP group, there was no significant difference in TSI‐SD between optimized LVSP and LVSP‐LVP. No significant difference in IVMD was observed between optimized LBBAP and LBBAP‐LVP or between LBBP and LVSP. Supporting Information: Figure S1 showed the LV mechanical synchrony evaluated by TSI in two patients during different pacing modes.

### 3.4 Clinical findings during follow‐up

**Figure 4:**
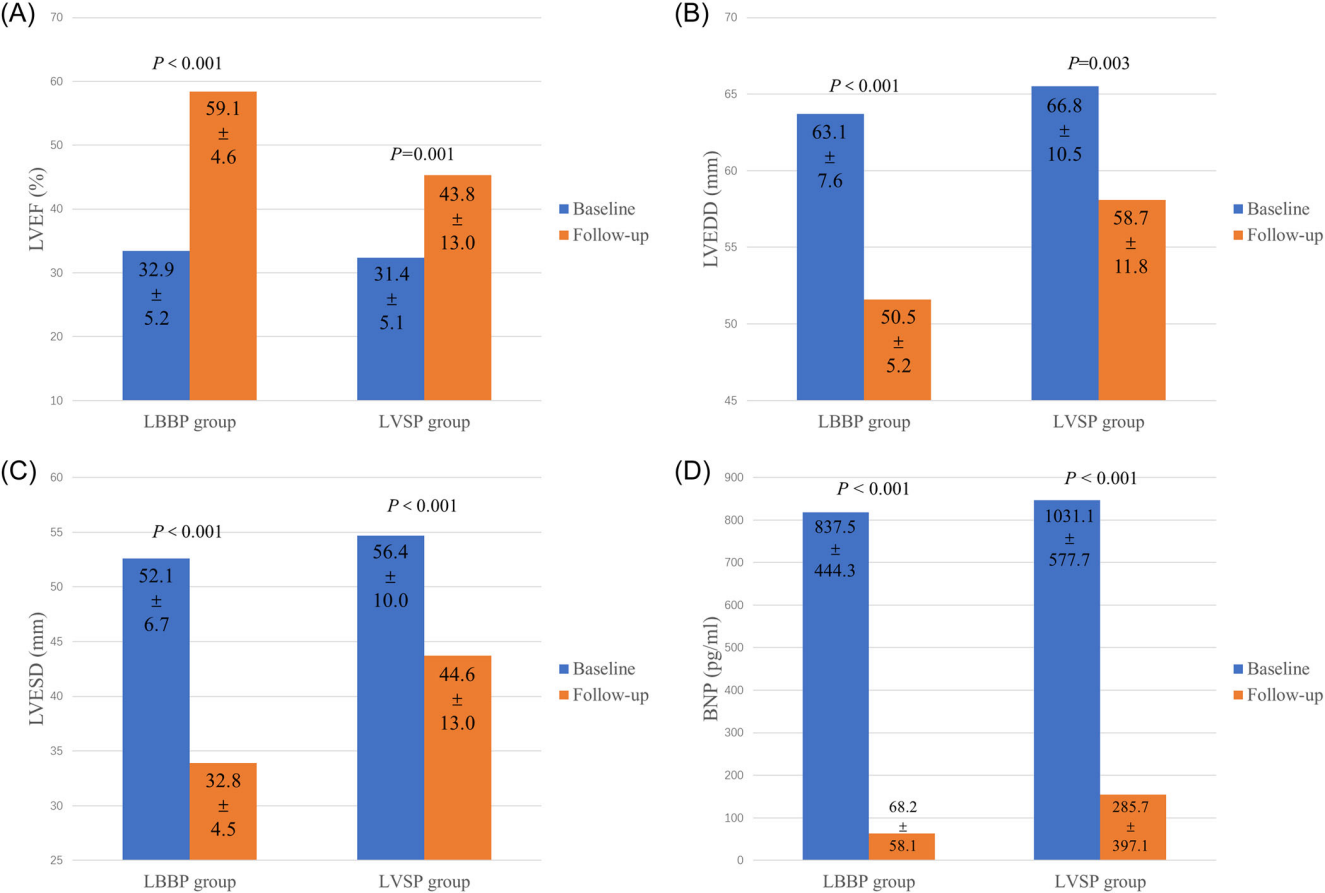
Clinical and echocardiographic assessments at baseline and during the follow‐up in the LBBP and LVSP groups. (A) Left ventricular ejection fraction (LVEF). (B) Left ventricular end diastolic dimension (LVEDD). (C) Left ventricular end systolic dimension (LVESD). (D) Plasma level of B‐type Natriuretic Peptides (BNP).  *p* values are for comparisons between baseline and follow‐up.

The average follow‐up time was 17 (IQR 8) months. The proportion of ventricular pacing was 99.4 ± 0.6% (range 98% to 100%). As shown in Figure 4, there were significant improvements in LVEF, LVEDD, LVESD, and plasma level of BNP in both groups at the last follow‐up. Patients also experienced significant improvements in NYHA classification and other echocardiographic measurements including LAD, MR and TR during follow‐up, except in the LBBP group where no significant improvement in TR was observed (Supporting Information: Figure S2).

Further analysis found that LVEF improved by 26.0% (IQR 16.0), LVEDD by 11.0 mm (IQR 9.0) and LVESD by 19.3 ± 5.8 mm in the LBBP group were significantly greater than those in the LVSP group (LVEF improved by 6.0% (IQR 20.8), LVEDD by 5.0 mm (IQR 12.5), LVESD by 11.8 ± 9.1 mm; *p* = .002, .044, and .013, vs. the LBBP group, respectively). In the LBBP group, all 15 patients (all with optimized LBBP) had CRT super response. Of the 14 patients in the LVSP group, 6 patients (3 optimized LVSP, 3 LVSP‐LVP) were super‐responders, 5 (1 optimized LVSP, 4 LVSP‐LVP) were moderate‐responders, and 3 (1 optimized LVSP, 2 LVSP‐LVP) were nonresponders (*p* = .001 compared with the LBBP group). Of the five patients with IVCD in the LVSP group, two had CRT moderate response and three were nonresponders.

At the last follow‐up, there was no significant difference in the use of standard medications for HF treatment in the two groups compared to those at baseline, except that the use of loop diuretics in the LBBP group was significantly reduced (*p* = .017 vs. baseline) (Supporting Information: Table S3). All patients had at least one hospitalization for HF in the 1‐year period before LBBAP. During follow‐up, no patients in the LBBP group were hospitalized for HF, and two patients (one nonresponder and one moderate‐responder) in the LVSP group experienced one HF hospitalization.

### 3.5 Pacing parameters and procedure‐related complications during follow‐up

There was no significant difference in LBBAP capture threshold between postimplantation and at follow‐up in both the two groups (Supporting Information: Table S4). No procedure related complications such as IVS perforation, lead dislocation, and obvious lead displacement were observed.

## 4 DISCUSSION

The present study demonstrated a significant improvement in cardiac function and favorable clinical effects of LBBAP in patients with HF and CRT indications, which is consistent with previous findings.^10^, ^11^, ^12^ Furthermore, the present study has several new findings. First, the study utilized intracardiac EGM to assess LV conduction time (CT) during pacing in addition to using LVAT and ECG morphology to further confirm LBBP and LVSP. The activation in the LV during LBBP propagated faster than that during LVSP. Second, LBBP provided better LV electrical and mechanical synchrony than LVSP. Third, the study found that the CRT response rate and the improvement of LVEF in the LBBP group were greater than those in the LVSP group. Poor improvement in LVEF was mainly found in the patients with IVCD.

Procedure endpoints of LBBAP include LBBP and LVSP,^13^ which were also confirmed in this study. Because LVSP is an acceptable result of failed LBBP implantation or local lead tip displacement at follow‐up, it remains impractical to compare the resynchronization effect and clinical outcomes of LBBP with LVSP in a randomized fashion. In addition, the term of LBBAP has been adopted and the LBBP and LVSP share similar ECG characteristics at a certain level. Therefore, the difference in resynchronization therapy between LBBP and LVSP in HF patients at relatively long‐term follow‐up is still unknown. The present study utilized a practical technique of LV CT measurement to differentiate LBBP from LVSP, especially during follow‐up. As a result, we were able to compare clinical outcomes between LBBP and LVSP, the two pacing modalities of LBBAP.

### 4.1 Differences in electrical and mechanical synchrony and clinical effects between LBBP and LVSP

In the present study, we developed a unique technique using intracardiac EGMs recorded by the LBBAP lead and the LV CS lead to measure the LV CT between the two sites during pacing. The study found that the CT during LBBP was significantly shorter than that during LVP, suggesting the recruitment of the conduction system during LBBP while LVP generates activation that propagates slowly via myocardial tissue. On the other hand, the CT during LVSP was similar to that during LVP, suggesting that both have a slower activation propagation. By using paced QRS morphology, LVAT and LV CT, we were able to confirm LBBP and LVSP during follow‐up and compare the clinical effects of LBBP and LVSP. Our findings in LV CT and ECG LVAT are consistent with previous findings that LBBP generates better LV electrical synchrony than LVSP.^4^, ^14^, ^15^

In the study by Hou et al.^15^ using single‐photon emission computed tomography myocardial perfusion imaging to evaluate LV mechanical synchrony of LBBAP, it was found that LV mechanical synchrony in patients with a LBB potential were better than those without a LBB potential, suggesting that LBBP has better LV mechanical synchrony than LVSP. The present study also showed better LV synchrony during LBBP than that during LVSP though both LBBP and LVSP generated better LV mechanical synchrony than during intrinsic rhythm. Both LBBP and LVSP, whether combined with LVP or not, had similar improved IVMD, implying that the differences in clinical effects by different LBBAP modes were mainly due to the differences in LV synchrony.

Superior LV electrical and mechanical synchrony during LBBP may explain the results of the present study that showed a better improvement in LVEF and a higher super response rate with LBBP than with LVSP. Better implantation tools and techniques should be developed to achieve a high success rate of LBBP.

### 4.2 Achieving optimized LBBP and LVSP

In the LBBP group, optimized LBBP was utilized in all patients by choosing an appropriate AV delay, which resulted in super response to CRT in all patients. This result was likely due to the restoration of the native LV conduction system and physiological LV synchrony, as well as the fusion of LV activation with the native right ventricular activation. Surprisingly, our study found that the LV TSI‐SD during LBBP appeared better than LBBP‐LVP, suggesting that adding LVP to LBBP (LOT‐CRT^12^) may not be needed in patients with LBBB. This is because LBBP can fully restore the functionality of the LBB. In patients with LVSP (the LVSP group), pacing options include (1) optimized LVSP, or (2) LVSP‐LVP which could achieve further shortening of QRSd in some patients. The present study found that some patients with optimized LVSP could also experience super response. The explanations may include (1) LVSP with a relatively narrow QRSd and short LVAT may have a delayed recruitment of the conduction system or (2) the slow conduction through IVS in patients with LBBB could be bypassed by LVSP.^16^ The study also observed that some of these patients with LVSP‐LVP still showed only moderate or no CRT response, especially in the patients with IVCD. In the His‐SYNC study, His‐CRT did not show significant advantage over biventricular pacing‐CRT (BiVP‐CRT).^17^ One of the main reasons was that His‐CRT could not obtain resynchronization in patients with IVCD. IVCD suggests not only diffuse intraventricular conduction disturbance, but also myocardial disease. CRT for patients with IVCD still needs investigations. Prospective, randomized studies in a large and broad HF population should be conducted to determine the superiority of optimized LBBAP or LBBAP‐LVP over BiVP‐CRT in patients with CRT indications.

## 5 LIMITATIONS

First, this is a single‐center, observational study. The comparison between LBBP versus LVSP in a nonrandomized fashion with a small sample size could lead to inclusion bias, which could also have an impact on the comparison of the baseline data and CRT response rate of the two groups. HF patients with LBBB and IVCD were all enrolled in the present study, which made the study cohort some heterogenous. While the present study used the existing LBBP criteria and new LV conduction time measurement to differentiate LBBP from LVSP, further validation in more patients is needed. Moreover, an additional LV pacing lead doesn't seem to be necessary for patients with direct LBB capture and LBBP‐LVP was not performed. However, because BiVP is recommended by current guidelines for patients with CRT indications and the safety and effectiveness of LBBP need to be further verified. Therefore, LV pacing lead was implanted as a backup. Although the present study showed that the electrical and mechanical resynchronization of LBBP‐LVP was not superior to that of optimized LBBP, further investigations are needed to determine whether LBBP‐LVP can provide additional therapeutic benefits. Besides, the success rate of LBBP in this study was around 60%, which might be due to the enrollment of patients with IVCD in the study cohort and the limitation of the number of lead screw‐in attempts.

## 6 CONCLUSIONS

The present study found that LBBP had superior electrical and mechanical synchrony and clinical effects over LVSP in HF patients with CRT indications. Importantly, the present study utilized the measurement of LV conduction time, which can enhance the current LBBP criteria to differentiate LBBP from LVSP. Furthermore, the study suggests that optimized LBBP is an effective CRT in HF patients with LBBB in whom LBBP‐LVP may not be necessary. Moreover, LVSP can be an alternative when LBBP cannot be achieved, and LVSP‐LVP should be considered if LVSP‐LVP generates narrower QRSd.

## CONFLICT OF INTEREST

Xiaohong Zhou is an employee of Medtronic, Inc. The remaining authors declare no conflict of interest.

## Supporting information

Supporting information.

Supporting information.

## Data Availability

Data available on request from the authors.
