# Supplementary material for: Resynchronization effects and clinical outcomes during left bundle branch area pacing with and without conduction system capture
Source: Clin Cardiol. 2023 Jan 3;46(3):287–95. doi: 10.1002/clc.23969 (PMC10018083; doi:10.1002/clc.23969)

**Supplemental Figures**

**Figure S1. Left intraventricular (LV) mechanical synchrony evaluated by tissue synchronization imaging (TSI) in different pacing modes.** The lower standard deviation of TSI of 12 LV segments (TSI-SD) indicates better LV mechanical synchrony. The colour coding ranges from green (the early activated segments), yellow, orange to red (the latest activated segments). One patient in the LBBP group had a TSI-SD of 12 ms during optimized LBBP (A), 35ms during LBBP-LVP (B) and 62 ms in the intrinsic rhythm (C). Another patient in the LVSP group had a TSI-SD of 53 ms during optimized LVSP (D), 45ms during LVSP-LVP (E) and 65 ms in the intrinsic rhythm (F).

**
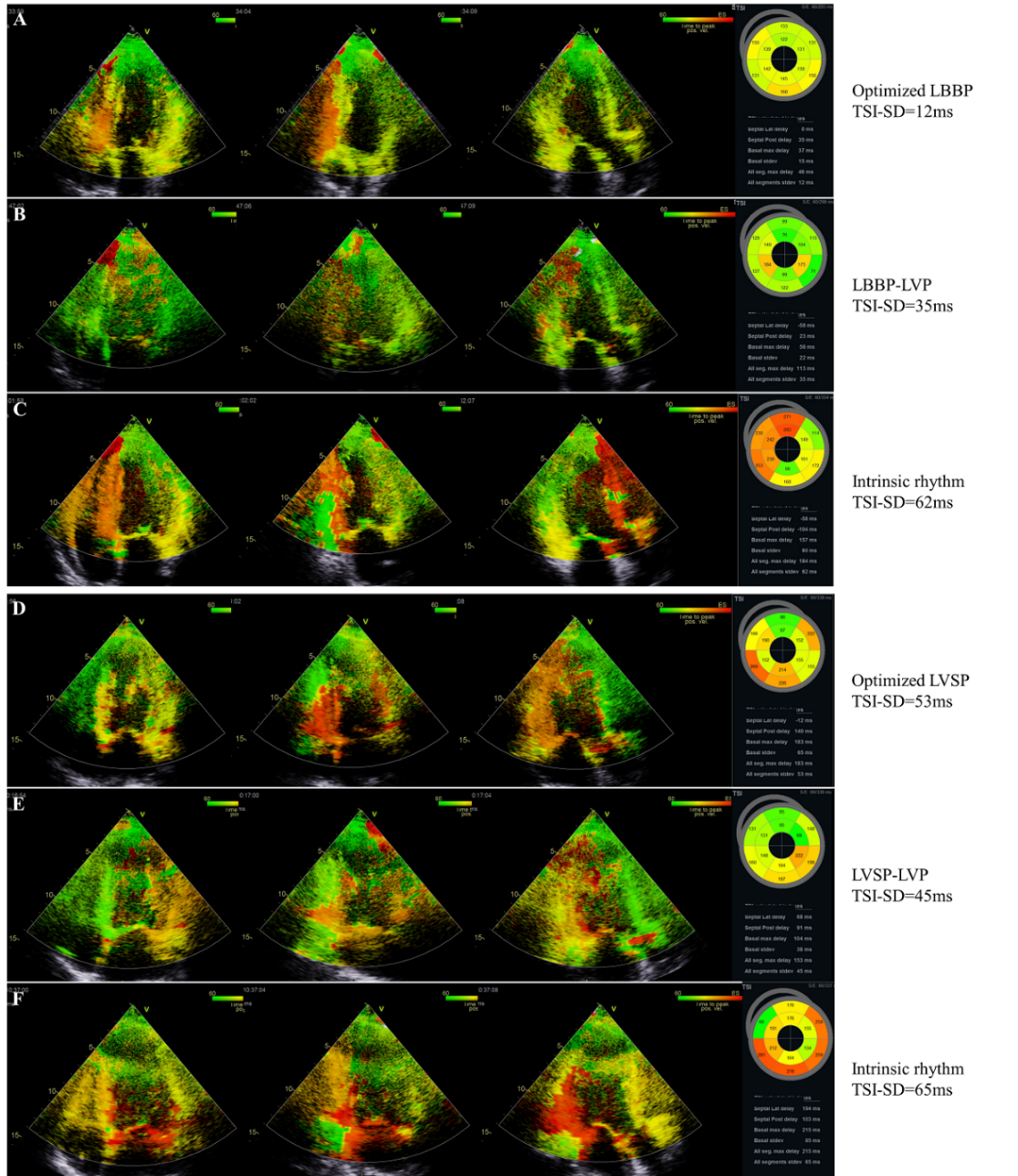
**

**Figure S2. Clinical and echocardiographic assessments at baseline and during the follow-up in the LBBP and LVSP groups.** A. New York Heart Association (NYHA) functional class. B. Left atrial dimension (LAD). C. Degree of mitral valve regurgitation (MR). D. Degree of tricuspid valve regurgitation (TR). *P*-values are the comparisons between baseline and follow-up.


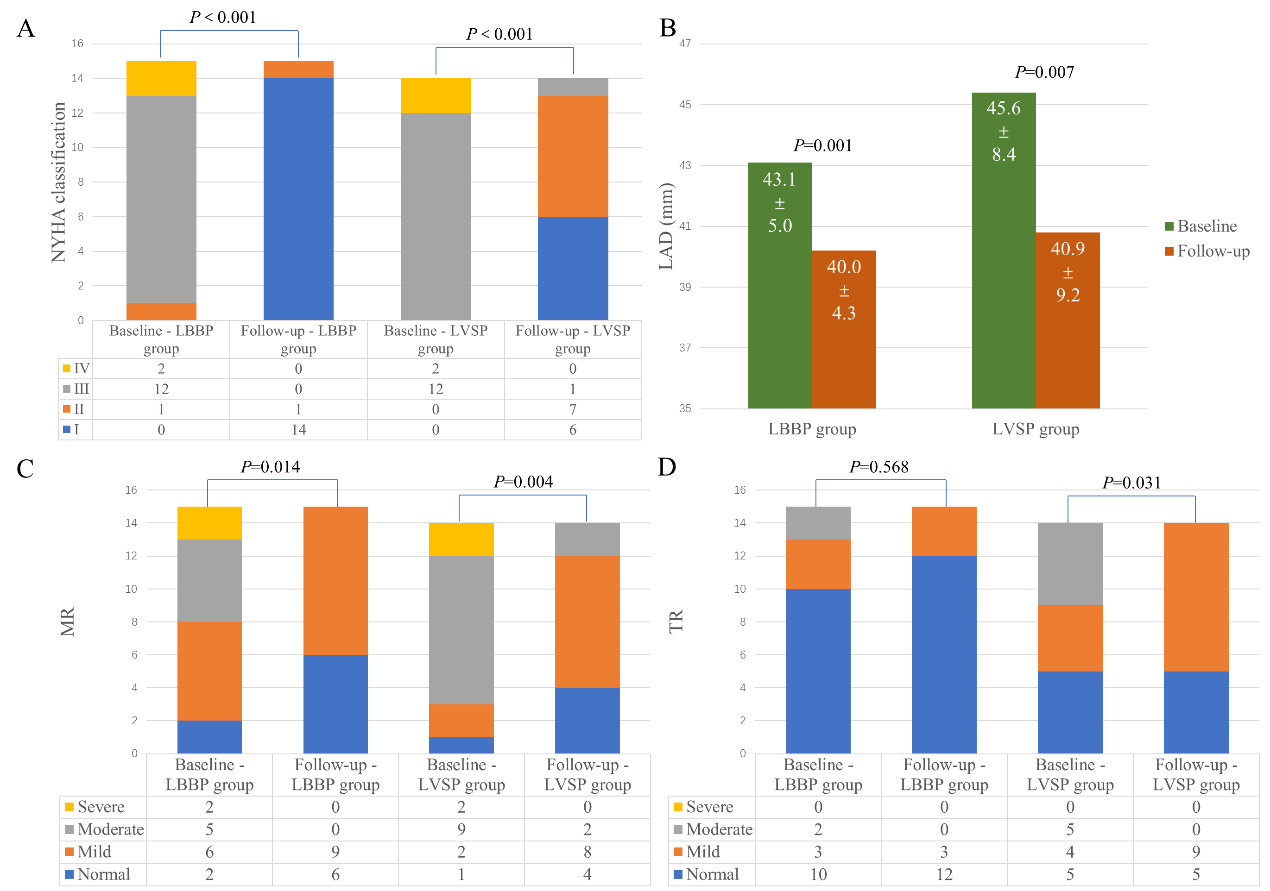

Supplement: Supplementary file 1 — Supporting information. [file CLC-46-287-s002.docx]
