# Supplementary material for: Resynchronization effects and clinical outcomes during left bundle branch area pacing with and without conduction system capture
Source: Clin Cardiol. 2023 Jan 3;46(3):287–95. doi: 10.1002/clc.23969 (PMC10018083; doi:10.1002/clc.23969)
Supplement: Supplementary file 2 — Supporting information. [file CLC-46-287-s001.docx]

**Supplemental Tables**

**Table S1.** Baseline characteristics of the patients for LBBAP.

|  | All patients  (N=29) | LBBP-group  （N=15) | LVSP-group  (N=14) | P-value |
| --- | --- | --- | --- | --- |
| Age (year) | 68.3±9.8 | 66.1±11.0 | 70.8±8.1 | 0.201 |
| Gender |  |  |  | 0.858 |
| Male, n (%) | 14 (48.3%) | 7（46.7%） | 7（50.0%） |  |
| Female, n (%) | 15 (51.7%) | 8（53.3%） | 7（40.0%） |  |
| ICM, n (%) | 4 (13.8%) | 1（6.7%） | 3（21.4%） | 0.330 |
| non-ICM, n (%) | 25 (86.2%) | 14（93.3%） | 11（78.6%） | 0.330 |
| CAD, n (%) | 8 (27.6%) | 4（26.7%） | 4（28.6%） | 1.000 |
| MI history, n (%) | 4 (13.8%) | 1（6.7%） | 3（21.4%） | 0.330 |
| AF, n (%) | 6 (20.7%) | 2（13.3%） | 4（28.6%） | 0.390 |
| VHD, n (%) | 1 (3.4%) | 0（0%） | 1（7.1%） | 0.483 |
| CRT indications |  |  |  |  |
| LBBB, n (%) | 24 (82.8%) | 15（100.0%） | 9（64.3%） | 0.017 |
| IVCD, n (%) | 5 (17.2%) | 0（0%） | 5（35.7%） | 0.017 |
| Intrinsic QRSd (ms) | 171.9±14.5 | 169.0±13.3 | 174.9±15.6 | 0.280 |
| Intrinsic LVAT (ms) | 106.5±25.0 | 102.6±17.5 | 110.6±31.3 | 0.407 |
| Echocardiographic indices |  |  |  |  |
| LVEF (%) | 32.2±5.1 | 32.9±5.2 | 31.4±5.1 | 0.418 |
| LVEDD (mm) | 64.9±9.1 | 63.1±7.6 | 66.8±10.5 | 0.282 |
| LVESD (mm) | 54.2±8.5 | 52.1±6.7 | 56.4±9.9 | 0.188 |
| LAD (mm) | 44.3±6.9 | 43.1±5.0 | 45.6±8.4 | 0.336 |
| MR |  |  |  | 0.385 |
| none, n (%) | 3 (10.3%) | 2（13.3%） | 1（7.1%） |  |
| mild, n (%) | 8 (27.6%) | 6（40.0%） | 2（14.3%） |  |
| moderate, n (%) | 14 (48.3%) | 5（33.3%） | 9（64.3%） |  |
| severe, n (%) | 4 (13.8%) | 2（13.3%） | 2（14.3%） |  |
| TR |  |  |  | 0.231 |
| none, n (%) | 15 (51.7%) | 10（66.7%） | 5（35.7%） |  |
| mild, n (%) | 7 (24.1%) | 3（20.0%） | 4（28.6%） |  |
| moderate, n (%) | 7 (24.1%) | 2（13.3%） | 5（35.7%） |  |
| severe, n (%) | 0 | 0 | 0 |  |
| NYHA classification |  |  |  | 1.000 |
| I, n (%) | 0 | 0 | 0 |  |
| II, n (%) | 1 (3.4%) | 1（6.7%） | 0 |  |
| III, n (%) | 24 (82.8%) | 12（80.0%） | 12（85.7%） |  |
| IV, n (%) | 4 (13.8%) | 2（13.3%） | 2（14.3%） |  |
| BNP (pg/ml) | 931.0±513.2 | 837.5±444.3 | 1031.1±577.7 | 0.319 |
| Hospitalization for HF in 1 year period before LBBAP | 2.00（1.00） | 2.00（1.00） | 2.00（1.00） | 0.799 |
| HF-related medications |  |  |  |  |
| ARNI, n (%) | 18 (62.1%) | 10（66.7%） | 8（57.1%） | 0.597 |
| ACEI/ARB, n (%) | 11 (37.9%) | 5（33.3%） | 6（42.9%） | 0.597 |
| β-blocker, n (%) | 28 (96.6%) | 15（100.0%） | 13（92.9%） | 0.483 |
| Spironolactone, n (%) | 29 (100.0%) | 15（100%） | 14（100%） | - |
| Diuretics, n (%) | 29 (100.0%) | 15（100%） | 14（100%） | - |

AF= atrial fibrillation, ACEI= angiotensin converting enzyme inhibitor, ARB= angiotensin receptor blocker, ARNI= angiotensin receptor neprilysin inhibitor, BNP= B-type natriuretic peptides, CAD= coronary artery disease, CRT= Cardiac resynchronization therapy, HF= heart failure, ICM= ischemic cardiomyopathy, IVCD= intraventricular conduction disturbance, LAD= left atrial dimension, LBBAP= left bundle branch area pacing, LBBB= left bundle branch block, LBBP= left bundle branch pacing, LVAT= peak left ventricular activation time, LVEDD= left ventricular end diastolic dimension, LVEF= left ventricular ejection fraction, LVESD= left ventricular end systolic dimension, LVSP= left ventricular septal pacing, MI= myocardial infarction, MR= mitral valve regurgitation, non-ICM= non-ischemic cardiomyopathy, NYHA= New York heart association, QRSd= QRS duration, RBBB= right bundle branch block, TR= tricuspid valve regurgitation, VHD= valvular heart disease. *P*-values were the comparisons between the LBBP group and LVSP group.

**Table S2. The electrical characteristics and echocardiographic mechanical** **synchrony in the LBBP group and LVSP group.**

|  | LBBP group | | |  | LVSP group | | | *P*-value^a^ |
| --- | --- | --- | --- | --- | --- | --- | --- | --- |
|  | Intrinsic rhythm (N=15) | Optimized LBBP (N=15) | LBBP-LVP (N=13) |  | Intrinsic rhythm (N=14) | Optimized LVSP (N=14) | LVSP-LVP (N=14) |  |
| QRSd (ms) | 169.0±13.3 | 128.8±16.6 | 126.6±16.8 |  | 174.9±15.6 | 149.1±18.9 | 136.3±19.3 | 0.005 |
| LVAT (ms) | 102.6±17.5 | 71.2±8.3 | 73.2±9.0 |  | 110.6±31.3 | 89.1±19.5 | 78.8±17.1 | 0.005 |
| TSI-SD (ms) | 58.1±13.6 | 35.3±9.5 | 43.2±16.5 |  | 65.6±11.8 | 49.8±14.4 | 51.1±11.7 | 0.003 |
| IVMD (ms) | 49.8±14.5 | 20.1±9.2 | 17.5±9.1 |  | 61.1±31.9 | 26.6±20.4 | 28.4±20.6 | 0.284 |

IVMD= inter-ventricular mechanical delay, TSI-SD= the standard deviation of tissue synchronization imaging (TSI) of 12 LV segments. *^a^ P*-values were the comparisons between the optimized LBBP in the LBBP group and the optimized LVSP in the LVSP group.

**Table S3.** The use of standard medications for HF treatment in the two groups at baseline and at the last follow-up.

|  | At the last follow-up | | |  | Before LBBAP procedure (At baseline) | | | |
| --- | --- | --- | --- | --- | --- | --- | --- | --- |
|  | LBBP group  (N=15) | LVSP group  (N=14) | *P*-value^a^ |  | LBBP group  (N=15) | P-value^b^ | LVSP group  (N=14) | *P*-value^c^ |
| HF-related medications |  |  |  |  |  |  |  |  |
| ARNI, n (%) | 11（73.3%） | 12（85.7%） | 0.651 |  | 10（66.7%） | 1.000 | 8（57.1%） | 0.209 |
| ACEI/ARB, n (%) | 4（26.7%） | 2（14.3%） | 0.651 |  | 5（33.3%） | 1.000 | 6（42.9%） | 0.209 |
| β-blocker, n (%) | 15（100.0%） | 14（100.0%） | - |  | 15（100.0%） | - | 13（92.9%） | 1.000 |
| Spironolactone, n (%) | 15（100%） | 14（100%） | - |  | 15（100%） | - | 14（100%） | - |
| Diuretics, n (%) | 9（60.0%） | 12（85.7%） | 0.215 |  | 15（100%） | 0.017 | 14（100%） | 0.481 |

^a^ *P*-values were the comparisons between the LBBP group and LVSP group at the last follow-up. ^b^ *P*-values were the comparisons of the medications use at the last follow-up and baseline in the LBBP group. ^c^ *P*-values were the comparisons of the medications use at the last follow-up and baseline in the LVSP group.

**Table S4.** The LBBAP capture threshold in the two groups at the post-implantation and at the last follow-up.

|  | LBBP group （N=15） | | |  | LVSP group （N=14） | | |
| --- | --- | --- | --- | --- | --- | --- | --- |
|  | Post-implantation | At follow-up | *P*-value |  | Post-implantation | At follow-up | *P*-value |
| LBBAP capture threshold （V/0.4ms） | 0.92±0.46 | 0.88±0.43 | 0.290 |  | 0.76±0.12 | 0.71±0.20 | 0.355 |

*P*-values were the comparisons of the LBBAP capture threshold between post-implantation and at follow-up in the two groups.
